# Survival of Plants During Short-Term BOA-OH Exposure: ROS Related Gene Expression and Detoxification Reactions Are Accompanied With Fast Membrane Lipid Repair in Root Tips

**DOI:** 10.1007/s10886-021-01337-z

**Published:** 2022-01-05

**Authors:** Laura Laschke, Vadim Schütz, Oliver Schackow, Dieter Sicker, Lothar Hennig, Diana Hofmann, Peter Dörmann, Margot Schulz

**Affiliations:** 1 IMBIO Institute of Molecular Physiology and Biotechnology of Plants, University of Bonn, Karlrobert-Kreiten Str. 13, 53115 Bonn, Germany; 2 Institute of Organic Chemistry, Institut Für Organische Chemie, Universität Leipzig, Johannisallee 29, 04103 Leipzig, Germany; 3 IBG-3: Agrosphäre, Forschungszentrum Jülich GmbH, Jülich, Germany

**Keywords:** BOA-OH isomers, ROS, Detoxification, *FAD2-2*, *SOD2*, Lipids, Membrane repair

## Abstract

For the characterization of BOA-OH insensitive plants, we studied the time-dependent effects of the benzoxazolinone-4/5/6/7-OH isomers on maize roots. Exposure of *Zea mays* seedlings to 0.5 mM BOA-OH elicits root zone-specific reactions by the formation of dark rings and spots in the zone of lateral roots, high catalase activity on root hairs, and no visible defense reaction at the root tip. We studied BOA-6-OH- short-term effects on membrane lipids and fatty acids in maize root tips in comparison to the benzoxazinone-free species *Abutilon theophrasti* Medik. Decreased contents of phosphatidylinositol in *A. theophrasti* and phosphatidylcholine in maize were found after 10–30 min. In the youngest tissue, α-linoleic acid (18:2), decreased considerably in both species and recovered within one hr. Disturbances in membrane phospholipid contents were balanced in both species within 30–60 min. Triacylglycerols (TAGs) were also affected, but levels of maize diacylglycerols (DAGs) were almost unchanged, suggesting a release of fatty acids for membrane lipid regeneration from TAGs while resulting DAGs are buildings blocks for phospholipid reconstitution, concomitant with BOA-6-OH glucosylation. Expression of superoxide dismutase (*SOD2*) and of ER-bound oleoyl desaturase (*FAD2-2*) genes were contemporaneously up regulated in contrast to the catalase *CAT1,* while *CAT3* was arguably involved at a later stage of the detoxification process. Immuno-responses were not elicited in short-terms, since the expression of *NPR1, POX12* were barely affected, *PR4* after 6 h with BOA-4/7-OH and *PR1* after 24 h with BOA-5/6-OH. The rapid membrane recovery, reactive oxygen species, and allelochemical detoxification may be characteristic for BOA-OH insensitive plants.

**Supplementary Information:**

The online version contains supplementary material available at 10.1007/s10886-021-01337-z.

## Introduction

An increase of reactive oxygen species (ROS) is a characteristic feature when plants are exposed to their own allelochemicals or those from other plants (Gniazdowska and Bogatek 2005; Lara-Nuñez et al. 2006; Huang et al. 2020). H_2_O_2_ and the superoxide anion radical (O_2_^**·−**^) are key compounds in signaling and cell wall loosening reactions (Baxter et al. 2014; Marino et al. 2012; Liszkay et al. 2004). However, excessive amounts of reactive oxygen species disturb the cellular ROS homeostasis. The imbalance leads to oxidative damage of biomolecules, starting with membrane lipids, which is thought to be one of the major reasons for reduced growth or even death of sensitive plant seedlings with insufficient detoxification capacities. When roots are exposed to solutions of allelochemicals, the plasma membrane is the first membrane system contacted. Membrane interactions of hydrophobic allelochemicals or those possessing hydrophobic domains are attributed to membrane leakage, one of the earliest and most harmful injuries caused by toxic allelochemicals (Schulz et al. 2013; Einhellig et al. 1986). For instance, cinnamaldehyde interacts with membrane receptors, whereas allelopathic concentrations of citronellal and citronellol disorganize membrane lipids (Lins et al. 2019), some allelochemicals change the membrane potential (Maffei et al. 2001). The allelochemical benzoxazolinone (BOA), for instance, induces oxidative stress in lettuce, mung bean, or maize, resulting in enhanced malondialdehyde (MDA) levels (Sanchez-Moreiras and Reigosa 2005; Batish et al. 2006; Schulz et al. 2013). The increases of MDA and conjugated dienes are indicative of lipid/ polyunsaturated fatty acid peroxidation. The loss of membrane functions is a consequence of lipid peroxidation and damaged enzymes anchored therein or otherwise attached (Stark 2005).

Although lipid peroxidation caused by allelochemicals was often postulated from increased MDA levels, there is no study addressing the effects on lipids with defined molecular species, and how fast, if at all, repair of membranes is initiated, a prerequisite to reestablish functional membranes. Indeed, prompt membrane regeneration is crucial for the survival of allelochemical effects and may be characteristic of tolerant plant species.

Benzoxazinones are secondary metabolites found in several grasses and some dicotyledonous species (Sicker et al. 2000; Niemeyer 2009). DIMBOA (2,4-dihydroxy-7-methoxy-2*H*-1,4-benzoxazin-3(4*H*)-one) and its glucosylated form is the major benzoxazinone in maize. DIMBOA but also DIBOA (2,4-dihydroxy-2*H*-1,4-benzoxazin-3(4*H*)-one) which is, for instance, present in rye and old maize leaves, possess biocidal properties, influence growth of plants and microorganisms, and can function as a toxin for sensitive herbivores (Schütz et al. 2019; Hu et al. 2018; Maag et al. 2016; Wouters et al. 2016; Neal et al. 2012; Schulz et al. 2013).

Benzoxazolin-2(3*H*)-one is the first degradation product of DIBOA. Less sensitive plants can hydroxylate BOA in position 5 or, preferentially, in position 6, and immediate glucosylation yields BOA-5/6-*O*-glucoside as a minor or major detoxification product (Sicker and Schulz 2002; Fig. S1). Maize detoxifies BOA mainly via glucoside carbamate, whereas BOA-6-*O*-glucoside is a minor detoxification product occurring only during the early detoxification process. BOA detoxification via glucoside carbamate in maize roots is accompanied by pronounced shifting of proteins between different cellular compartments (Schulz et al. 2016). In contrast, BOA-6-*O*-glucoside and methoxylated glucoside carbamate represent major and minor products in roots, respectively, when maize seedlings are incubated with MBOA (Hofmann et al. 2006). BOA-6-*O*-glucoside can be detected occasionally in maize roots in low amounts without previous BOA incubation (Schulz et al. 2013). The more toxic intermediate BOA-6-OH does not accumulate in maize but often in seedlings of sensitive dicotyledonous species (Sicker et al. 2004). In contrast, the weed *Abutilon theophrasti* Medik. (velvetleaf), which is highly resistant to BOA when cultured in soils rich in organic matter, accumulates low amounts of BOA detoxification products, compared to maize, although roots of *A*. *theophrasti* can detoxify BOA via BOA-6-OH glucosylation by a cell wall associated glucosyltransferase (Haghi Kia et al. 2014). Instead, other strategies have been evolved to cope with BOA that includes root colonizing microorganisms, efficient transporters for BOA efflux, and numerous enzymes for BOA-6-OH polymerization and degradation at the root surface and within the apoplast (Schulz et al. 2012, 2018). A high catalase activity, indicated by bubble formation, was stated as a response to increased H_2_O_2_ at the root surface when *A. theophrasti* seedlings were exposed to BOA-OH isomers. Microorganisms colonizing the root surface, such as *Papiliotrema baii* and *Pantoea ananatis,* contribute dramatically to H_2_O_2_ generation, a concrete hint that microbial assemblies on the root surface suffer severely from the applied allelochemicals (Schulz et al. 2018). Therefore, membrane damage by lipid peroxidation may occur, at least during the early phase of allelochemical exposure.

In addition to the aforementioned BOA-6- and BOA-5-*O*-glucosides, BOA-4-*O*-glucoside was identified in benzoxazinone- (BXs) containing *Acanthus ilicifolius* plants (Huo et al. 2005). The isomer BOA-7-OH and its glucoside are not known as natural products. The detoxification behavior of crops, also that of BX containing plants such as maize, is of special interest for estimating the toxicity/ autotoxicity of these compounds because benzoxazinoids are increasingly in the focus for agricultural usage (Macias et al. 2005). For instance, BX-containing rye mulches are known to suppress certain weeds except for *A. theophrasti* in sustainable agricultural systems (Tabaglio et al. 2008; Boselli et al. 2021). Moreover, natural compounds with elicitor or priming properties for resistance and immunity of plants are under consideration as agents against diseases.

Maize root membranes contain the phospholipids (PLs) phosphatidylcholine (PC) as the dominant PL, phosphatidylethanolamine (PE), phosphatidylserine (PS), phosphatidylinositol (PI), phosphatidylglycerol (PG), the glycolipids (GLs) monogalactosyldiacylglycerol (MGDG), digalactosyldiacylglycerol (DGDG), and low amounts of free fatty acids. Plasma membranes of maize roots are composed predominately of PC and PE, whereas PG, PI, and PS are less abundant (Bohn et al. 2001). The glycolipid DGDG is a minor constituent of the plant plasma membrane, and it is located in the cytosolic leaflet (Tjellström et al. 2010; Cacas et al. 2016). PC and PE are also major lipids of mitochondria (Michaud et al. 2017). For *A. theophrasti*, no data on membrane lipid composition are available, but in all plants investigated, PLs and GLs are composed of different molecular species that vary in the lengths of the acyl chains and the number of double bonds. Within the apoplastic and the cytosolic leaflets of the plasma membrane, lipids are asymmetrically distributed, whereby the cytosolic leaflet has a higher content of unsaturated phospholipids. In vegetative tissue, triacylglycerols (TAGs) which are stored in lipid droplets in the cytosol, play a role in stress responses (Lu et al. 2020). Diacylglycerols (DAGs) can be synthesized from PA (phosphatidic acid). DAG is an intermediate metabolite in lipid metabolism and a precursor of PC, PE, PG, and PS. It can be acetylated to TAG, while hydrolysis of phospholipids or TAG by (phospho)lipases yield again DAG, representing also a signaling molecule. DAGs are in low concentration in the cytoplasmic leaflet of membranes but are found in higher amounts in the ER, mitochondria, and chloroplasts (Vermeer et al. 2017).

In this study, we describe the synthesis of four BOA-OH isomers as compounds causing oxidative stress in maize root tips, and time-dependent differentiated reactions of the root zones faced with these compounds. We looked for early impacts of BOA-6-OH on root membrane lipids of maize in comparison to the weed *A. theophrasti* and on membrane recovery by determination of total fatty acids and structural PLs, TAGs, and DAGs in maize and *Abutilon* seedlings. We set focus on the transient disturbance of lipid and fatty acid contents in the root tips when exposed to prevalent BOA-6-OH and the subsequent restoration of the lipid pools. With maize, the influence on superoxide dismutase (*SOD*) and catalase (*CAT*) gene expressions for ROS detoxification and on desaturase *FAD2* genes for unsaturated fatty acid regeneration in the youngest and most vulnerable root tissue exhibiting responses on the lipid level were studied. We searched for a coordination of membrane repair, ROS elimination, BOA-OH detoxification, and presumable links to pathogenesis and resistance in maize root tips. The study aimed to obtain better insights in the BOA-OH responses of maize known to be insensitive to BOA-6-OH and whether BOA-OH isomers differ in the time-dependent effects on maize.

## Methods and Materials

### Syntheses of Hydroxylated BOA Species

Except for BOA-6-OH, hydroxylated benzoxazolinones are commercially not available. Therefore, the isomers of hydroxylated benzoxazolinones, including BOA-6-OH, were synthesized to compare their detoxification in maize seedlings and to study gene expression. However, the laborious syntheses and yields of the different isomers did not allow to perform all experiments with all isomers.

2-Nitroresorcinol (**1**) was prepared by nitration of resorcinol with conc. H_2_SO_4_ and 67% HNO_3_. **1** was isolated by steam distillation as orange crystals (24% yield, 11 g). The melting points (mp) of 85 °C was in accordance with Schaffrath (1970). NMR spectra confirmed the structure: ^1^H-NMR (CDCl_3_, 300 MHz) δ 10.65 (2 H, s, O–H), 7.44 (1 H, t, *J* = 8.4 Hz, H-5), 6.61 (2 H, d, *J* = 8.4 Hz*,* H-4_,_ H-6). ^13^C-NMR (CDCl_3,_ 75 MHz) δ 156.5 (C, C-1, C-3). 138.9 (CH, C-5), 123.9 (C, C-2), 109.6 (CH, C-4, C-6).

2-Nitrohydroquinone (**2**) was prepared by the classical Elbs oxidation of 2-nitrophenol with K_2_S_2_O_8_ in diluted NaOH solution (Elbs 1893). **2** was obtained as dark red crystals in 9% yield and 3 g amount. The purification was done by removal of unreacted 2-nitrophenol by steam distillation followed by extraction of **2** from the residue with MTBE (methyl *t*-butyl ether) and a final crystallization of the MTBE extract from water. The mp of 131–132 °C is in accordance with the data of Elbs (1893). ^1^H-NMR (DMSO-d_6_, 300 MHz) δ10.14 (1 H, brs, O–H), 9.60 (1 H, brs, O–H), 7.22 (1 H, d, *J* = 2.9 Hz*,* H-3),7.02 (1H, dd, *J* = 9.0, 2.9 Hz, H-5), 6.96 (1 H, d, *J* = 9.0 Hz, H-6). ^13^C-NMR (DMSO-d_6,_ 75 MHz) δ149.7 (C, C-4), 145.1 (C, C-1), 135.8 (C, C-2), 124.3 (CH, C-5), 120.1 (CH, C-6), 109.5 (CH, C-3).

4-Nitroresorcinol (**3**) was prepared from resorcinol according to the recent nitration method of Samajdar et al. (2000) with silica gel-supported Bi(NO_3_)_3_ × 5 H_2_O with some variations (silica gel instead of montmorillonite, no microwave irradiation, CH_2_Cl_2_ as solvent during the reaction with the supported reagent). **3** was isolated by extraction with CHCl_3_ and purified by flash chromatography over silica gel (eluent *n*-hexane/ethyl acetate 2:1 v/v) as yellow crystals in 26% yield and 2 g amount. The mp of 120 °C is in accordance with Kauffmann and Kugel (1911). ^1^H-NMR (DMSO-d_6_, 300 MHz) δ 10.95 (1 H, brs, O–H), 10.80 (1 H, brs, O–H), 7.88 (1 H, d, *J* = 9.1 Hz*,* H-5), 6.42 (1H, d, *J* = 2.4 Hz, H-6), 6.39 (1 H, dd, *J* = 9.1, 2.4 Hz, H-2). ^13^C-NMR (DMSO-d_6,_ 75 MHz) δ 164.9 (C, C-1), 156.0 (C, C-3), 128.1 (C, C-4), 127.8 (CH, C-5), 108.8 (CH, C-6), 103.6 (CH, C-2).

3-Nitrocatechol (**4**) was prepared by nitration of an ethereal solution of catechol with 100% fuming nitric acid according to Rosenblatt et al. (1953). **3** was separated from the accompanying 4-nitrocatechol by steam distillation as dark yellow crystals in 15% yield and 4 g amount. The mp of 87 °C was in accordance with the literature (Samajdar et al. 2000). ^1^H-NMR (CDCl_3_, 300 MHz) δ10.62 (1 H, brs, O–H), 7.65 (1 H, dd, *J* = 8.7, 1.5 Hz, H-4), 7.24 (1 H, dd, *J* = 8.4, 1.2 Hz*,* H-6), 6.91 (1H, dd, *J* = 8.7, 8.4 Hz, H-5), 5.82 (1 H, brs, O–H). ^13^C-NMR (CDCl_3,_ 75 MHz) δ 146.5 (C, C-1), 142.8 (C, C-2), 133.8 (C, C-3), 121.7 (CH, C-5), 119.8 (CH, C-6), 115.8 (CH, C-4).

### General Method for the Synthesis of the Hydroxy-Substituted BOAs *5–8*.

In a glass hydrogenation flask, 1.54 g (9.9 mmol) of the corresponding nitro compound (compounds **1–4**) were dissolved in 150 ml of dry THF followed by 0.5 g Pd/C catalyst. The mixture was stirred under hydrogen from a balloon at standard pressure for 6 h. The H_2_ balloon was then removed, the flask was cooled in an ice water bath and 2.3 g (22 mol; 3.0 ml) triethylamine was added at once followed by dropwise addition of 0.8 g (2.6 mmol) triphosgene dissolved in 50 ml dry THF over a dropping funnel within 3 min. The mixture was stirred for 30 min. Then, the catalyst and the triethylamine hydrochloride were filtered. The solvent was removed from the filtrate in vacuo. The remaining residue was purified by column chromatography using CH_2_Cl_2_/methanol 10:1 as the eluent. An exception is compound **7**. All melting points given here were in accordance with those reported by Zinner and Wigert (1960), who used a different urea-based synthesis. The structures were further confirmed by ^1^H- and ^13^C-NMR, IR, and MS. NMR analyses were done with DRX-400 and Fourier 300 instruments (Bruker). The IR-spectra were obtained with a Fourier Transform Infrared Spectrometer FT/IR-4100 (JASCO). The EI-MS data were acquired on a MAT 8230 spectrometer (Thermo Fisher, formerly Finnigan MAT). Melting points were determined with a Boëtius micro hot stage.

4-Hydroxybenzoxazolin-2(3*H*)-one **5:** Yield: 998 mg (66%) off-white crystals, mp. 288–289 °C (Zinner and Wigert 1960: 281–283 °C). ^1^H-NMR (DMSO-d_6_, 300 MHz) δ10.69 (2 H, brs, O–H, N–H), 6.86 (1 H, t, *J* = 8.2 Hz, H-6), 6.71 (1 H, d, *J* = 8.2 Hz*,* H-7), 6.63 (1 H, d, *J* = 8.2 Hz, H-5). ^13^C-NMR (DMSO-d_6,_ 75 MHz) δ 154.5 (C, C-2), 144.7 (C, C-7a), 142.0 (C, C-4), 122.0 (CH, C-6), 118.2 (C, C-3a), 110.9 (CH, C-5), 100.9 (CH, C-7). IR (KBr): $\stackrel{\sim }{\upnu }$ $[c{m}^{-1}]$ = 3449, 3287, 1738, 1476, 1024. EI-MS m/z (%): 151(M + , 100), 79 (29), 67 (30), 57 (60), 43 (39).

5-Hydroxybenzoxazolin-2(3*H*)-one **6:** Yield: 651 mg (43%) beige crystals, mp. 212–213 °C (Zinner and Wigert 1960: 209–210 °C). ^1^H-NMR (DMSO-d_6_, 300 MHz) δ 11.33 (1 H, brs, N–H), 9.36 (1 H, brs, O–H), 7.02 (1 H, d, *J* = 8.6 Hz*,* H-7), 6.47 (1H, d, *J* = 2.4 Hz, H-4), 6.41 (1 H, dd, *J* = 8.6, 2.4 Hz, H-6). ^13^C-NMR (DMSO-d_6,_ 75 MHz) δ155.1 (C, C-2), 154.1 (C, C-5), 136.3 (C, C-7a), 131.0 (C, C-3a), 109.9 (CH, C-7), 108.0 (CH, C-6), 97.4 (CH, C-4).IR (KBr): $\stackrel{\sim }{\upnu }$ $[c{m}^{-1}]$ = 3541, 3464, 3285, 1731, 1482, 1172. EI-MS m/z (%): 151(M + , 100), 95 (95), 68 (36), 67 (32), 41 (29).

6-Hydroxybenzoxazolin-2(3*H*)-one **7:** A black solid remained after removal of the solvent *in vacuo*. It was refluxed for 10 min along with 0.5 g activated charcoal powder and 200 ml of distilled water. It was then filtered and cooled to yield 250 mg (17%) of **7** as colorless crystals, mp 299 °C (Zinner and Wigert 1960: 288–292 °C). Due to the high mp, the solubility of **7** is poor and column chromatography was not advisable. ^1^H-NMR (DMSO-d_6_, 300 MHz) δ 11.24 (1 H, brs, N–H), 9.36 (1 H, brs, O–H), 6.86 (1 H, d, *J* = 8.5 Hz*,* H-4), 6.69 (1H, d, *J* = 2.2 Hz, H-7), 6.55 (1 H, dd, *J* = 8.5, 2.2 Hz, H-5). ^13^C-NMR (DMSO-d_6,_ 75 MHz) δ154.8 (C, C-2), 153.1 (C, C-6), 144.1 (C, C-7a), 122.3 (C, C-3a), 110.1 (CH, C-5), 109.9 (CH, C-4), 98.0 (CH, C-7). IR (KBr): $\stackrel{\sim }{\upnu }$ $[c{m}^{-1}]$ = 3458, 3214, 1734, 1499, 1106. EI-MS m/z (%): 151(M + , 100), 95 (87), 67 (39), 52 (46), 41 (31).

7-Hydroxybenzoxazolin-2(3*H*)-one **8:** Yield: 420 mg (29%) beige crystals, mp 236–238 °C (Zinner and Wigert, 1960: 232 °C). ^1^H-NMR (DMSO-d_6_, 300 MHz) δ 10.50 (2 H, brs, N–H, O–H), 6.90 (1H, dd, *J* = 8.4, 7.8 Hz, H-5), 6.56 (1 H, dd, *J* = 8.4, 1.1 Hz*,* H-4), 6.51 (1 H, dd, *J* = 7.8, 1.1 Hz, H-6). ^13^C-NMR (DMSO-d_6,_ 75 MHz) δ 154.5 (C, C-2), 140.9 (C, C-7), 131.8 (C, C-3a), 131.1 (C, C-7a), 124.2 (CH, C-5), 110.3 (CH, C-4), 110.9 (CH, C-6).

IR (KBr): $\stackrel{\sim }{\upnu }$ $[c{m}^{-1}]$ = 3360, 3261, 1768, 1383, 1150. EI-MS m/z (%): 151(M + , 100), 96 (47), 68 (36), 67 (40), 52 (33).

### Plant Material

Caryopses of *Zea mays* cultivar Cassila (KWS, Germany) were hydroponically grown for 7 days as described in Schulz et al. (2016). *A. theophrasti* was grown as described in Schulz et al. (2017).

For the analysis of detoxification products, the maize seedlings were incubated with the BOA-isomers for 24 h (3 seedlings/ 20 ml of 0.5 mM of one of the isomers in tap water). For analysis of BOA-6-*O*-glc accumulation during short-term exposure, seedlings were incubated 0, 10, 20, 30, 40, 50, and 60 min with 0.5 µM BOA-6-OH. Analysis and determination of the detoxification products were performed by HPLC using the system and the method described below. For preparation of 500 ml incubation solution, the isomers were pre-solved in 1 ml methanol and sonicated for 5 min before adding tap water. Incubations with 9 seedlings/60 ml were repeated at least 6 times for each BOA-OH isomer. Browning reactions were monitored after 30 min, 1 h, 6 h, and 24 h, bubble formation after 10 min, 30 min, 1 h, and 6 h. Root extracts were performed as described in Schulz and Wieland (1998). Peroxidase activity at living root surfaces was detected by bathing the roots (1 seedling / 15 ml Falcon tube) in 10 ml 0.1 mM acetate buffer pH 5.0 supplemented with 1.6 ml 2 mM ABTS (Roche Diagnostics, Germany) and 0.6 ml 15 mM H_2_O_2_ (Sigma-Merck, Germany). Sites with peroxidase activity immediately turned dark or black-green. Catalase activity elicited by BOA-6,5,4-OH was concluded from bubble formation according to Schellhorn and Stones (1992) and Wheelis (2008), presenting a non-destructive method.

### Benzoxazinoids in Root Hairs and their Wash Solutions

For the harvest of root hairs, maize was germinated on wet filter paper placed in Petri dishes. Approximately 1 mg root hairs from six 7-day-old maize seedlings were cut using a binocular microscope and a micro scissors and immediately collected in a cap with 500 µl 70% ice cold methanol (n = 12). From another six seedlings, root hair zones were washed with 500 µl water, and the wash solutions including the mucilage drops (see below) collected in pure methanol, yielding a 50% solution. Four wash solutions collected over a period of 4 h were combined for one sample. Root hair extracts and wash solutions were analyzed by HPLC/ DAD (Shimadzu, Germany), equipped with a RP-18 column (Macherey–Nagel, Düren, EC 250/4.6 Nucleodur 100–5 C18). Linear gradients were run with 0.01% formic acid/H_2_O and methanol within 35 min. For confirmation of the compounds identity, root hair extracts were subsequently analyzed by Ultra Performance Liquid Chromatography (UPLC)-electrospray (ESI)-mass spectrometry (Waters, Eschborn) as described in Schulz et al. (2016). Mucilage drops from the root tips of 6 seedlings were collected on ice with a syringe and analyzed by HPLC. Since only traces of the BXs were found, further wash solutions contained washes of root hair zone including the mucilage drops (n = 7).

### Lipid Analyses

For total fatty acids methyl ester (FAME) determinations, 35 root tips (0-2 cm, further named ZmRTa) /sample and 10 older root parts (2–4 cm, further named ZmRTb)/sample from maize and 13 root tips (AbRTa)/ sample and 7 older root parts (AbRTb) /sample from *A. theophrasti* were harvested without incubation and after 10, 20, 30, 40, 50 and 60 min incubations in 0.5 mM BOA-6-OH. For these experiments, BOA-6-OH was purchased from Merck-Sigma. The harvested root material from each incubation time was immediately treated with boiling water for 20 min, followed by 3 × repeated chloroform/methanol (1:2), (1 ml/sample) extractions. The resulting three portions of crude lipid extracts were combined and purified by the addition of 0.75 ml 0.3 M ammonium acetate. After centrifugation at 2000 g for 5 min, the lipid phase was dried under N_2_. When not directly analyzed, the extracts were stored at -20 °C until analysis. The extracted root material was dried overnight at 104 °C for dry weight determination. The described extraction method followed the procedure described by Siebers et al. (2018). The conversion of fatty acids into fatty acids methyl esters (FAMEs) was done according to Browse et al. (1986). For quantification pentadecanoic acid, (15:0) was added as internal standard to the samples. FAMEs were analyzed using an Agilent 7890 gas chromatograph with Supelco SP-2380 capillary column and a flame ionization detector (7890 Gas chromatograph (GC) with flame ionization detector (FID) Agilent, Böblingen (D) as described in Siebers et al. (2018). The oven temperature was 100 °C initially, increased to 160 °C at 25 °C min^−1^, and finally ramped to 220 °C at 10 °C min^−1^. Fatty acid peaks were identified using FAME standards (Sigma-Aldrich, Supelco® 37 Component FAME Mix).

For the measurement of phospholipids, glycolipids, phospholipid fatty acid (PLFA) analyses, and determinations of TAGs and DAGs, 4 maize root parts/sample (see above) or 7 root parts/sample of *A. theophrasti* were extracted as described. Lipid extraction and analysis were according to Bravo et al. (2017). Lipids were fractionated according to their polarity by solid phase extraction (Gasulla et al. 2013; Siebers et al. 2015; Schütz et al. 2021). The fractionated phospholipids were methanolized and converted into their methyl esters prior to measurement by GC (Browse et al. 1986). The contents and molecular species composition of phospholipids/glycolipids, TAGs, and DAGs were determined by direct infusion mass spectrometry on an Agilent Accurate Mass quadrupole time-of-flight (Q-TOF) mass spectrometer. Quantification of lipid molecular species was performed by MS/MS analysis.

### Gene Expression Studies

#### Choice of Genes

The selection of genes for comparative expression studies was restricted to *SOD2, CAT 1, CAT 3, FAD2-1, FAD2-2, PR1, PR4,* and *POX 12* because of the limited amounts of BOA-4, 5- and 7-OH available for the experiments. For the same reason, the time points for real-time PCR were restricted, but within the most reactive period during the incubation regarding early gene responses, starting polymerization, and bubble development. The set of genes was selected to give first clues on the oxidative stress elicitation by BOA-OH isomers, lipid repair, and possible relations to plant immune responses in the youngest root tissues, thought as a preliminary base for future in-depth investigations. Superoxide dismutase 2 (SOD2) involved in superoxide radical transfer to H_2_O_2_ was included as an indicator for ROS stress (Gond et al. 2015). Mitochondrial catalase (*CAT3*) gene is responsive to H_2_O_2_, reactive oxygen species, and xenobiotics. Transcripts were detected in the root, epicotyl, and leaves (Redinbaugh et al. 1990). *CAT1* was described to be expressed in roots, shoots, and scutellum of young seedlings (Scandalios et al. 1983). While the *CAT1* expression is responsive to oxidative stress, the protein is localized to the cytosol (Mylona et al. 2007). *CAT 2* expression was described to belight-responsivee and was therefore not included in the studies (Guan et al. 1996). Expression of members of the complex gene families with functions in the glutathione–ascorbate (GSH–ASC) cycle, such as ascorbate peroxidase producing H_2_O from H_2_O_2,_ could not be addressed in this study. The gene expression of two ER-bound oleoyl desaturases (FAD2-1 and FAD2-2) was studied in context with lipid restoration processes after BOA-6-OH exposure. The enzymes synthesize linoleic acid (18:2) mostly in non-photosynthetic tissue (Dar et al. 2017). The seed and bud-specific desaturase FAD2-1 was considered not to be responsive, but effects of allelochemicals on *FAD2-1* expression are not known. *FAD2-2* was described as lowly expressed during the entire life cycle of the plant. To study whether the BOA-OHs induce the expression of genes related to pathogenesis and resistance, induction of *POX 12, PR1, PR4,* and *NPR1* expression was studied (Sajad et al. 2018; Backer et al. 2019; Hemetsberger et al. 2012). Primers are given in Tab. S 1.

#### RNA Isolation, cDNA Synthesis, and qRT-PCR

7-day-old maize seedlings were incubated with 0.5 mM of the BOA-OH isomers for 30 min, 1 h, and 6 h, for *SOD2* expression, BOA-6/5-OH samples in addition for 24 h, for *FAD2-2* expression all isomers also for 24 h. 100 mg Root tips were cut (3 cm) and immediately frozen in liquid nitrogen until RNA was extracted. The material was homogenized with the Precellys® 24 / 24-Dual (PEQLAB Biotechnologie, Erlangen) and RNA was isolated with the NucleoSpin RNA Plant Kit (Macherey and Nagel, Düren) according to the instructions of the manufacturer. For cDNA preparation, the Thermo Scientific RevertAid First Strand cDNA Synthesis Kit was used. RT-PCR was performed with a 7300 real-time PCR system (Applied Biosystems, Darmstadt). The reaction mixture (20 µl) consisted of 4 µl H_2_O, 5 µl cDNA, 1 µl primer pair (working solution 10 pmol/µl) and 10 µl 5xEvaGreen (ROX)qPCR-Mix II (Bio-Budget Technologies, Krefeld, Germany). Gene expression was determined relative to the control plants and to *ZmActin* as reference gene. For evaluation of RT-qPCR results, the ΔΔct method was applied.

### Statistics

Bars, presented with SD, were established with data obtained from three biological replicates unless otherwise noted. Statistical analysis of all the data was done with the t-test. Data in the figures are presented as the mean ± standard deviation. Significant differences between samples and the controls are indicated: ∗ p < 0.05; ∗  ∗ p < 0.005; ∗  ∗  ∗ p < 0.0005.

## Results

### Synthesis of Hydroxybenzoxazolin-2(3H)-ones and their Precursors

**Fig. 1:**
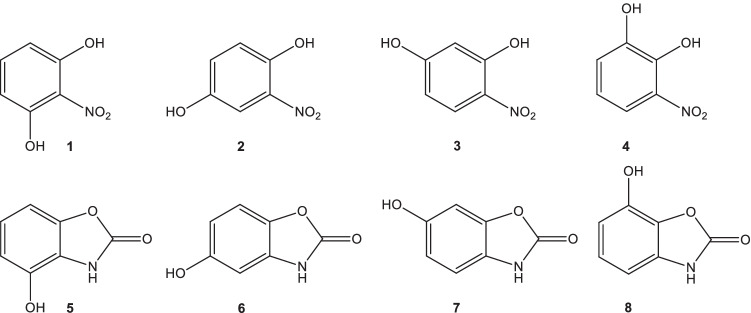
Nitro precursors **1–4** synthesized as precursors for the hydroxy BOAs **5–8**

All four hydroxy benzoxazolin-2(3*H*)-ones (**5**–**8**) were prepared from the corresponding nitro precursors **1**–**4** (Fig. 1) which, in turn, have been synthesized from resorcinol, hydroquinone, or catechol, respectively.

**Fig. 2:**
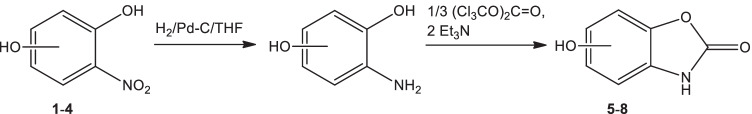
General Synthesis of the four isomeric hydroxy BOAs **5–8**

By hydrogenation of the nitro compounds in THF over Pd/C, the related amino compounds were obtained which have not yet been isolated in substance, due to their sensitivity towards oxygen. Instead, they have been directly subjected to CO insertion using triphosgene as the carbonyl source in the presence of triethylamine (Fig. 2). This method has first been described for the synthesis of the 6-methoxy analogue MBOA (Sicker 1989).

The solubility of the compounds in methanol decreased in the following order: BOA-4-OH > BOA-5-OH > BOA-6-OH > BOA-7-OH. The isomeric BOA-OHs were used for incubations of maize seedlings to reveal possible differences in the reaction of the root.

### Determination of Benzoxazinoid (BX) Contents in Cut Root Hair Extracts and Wash Solutions

To estimate benzoxazinoid concentrations which are released, but are not harmful to roots of the maize cultivar Cassila, the amounts of the major BX compounds, present in root hairs and wash solutions, were determined. These measurements verified that exudates did not contain detectable amounts of BOA-6-OH or its glucoside.

**Fig. 3:**
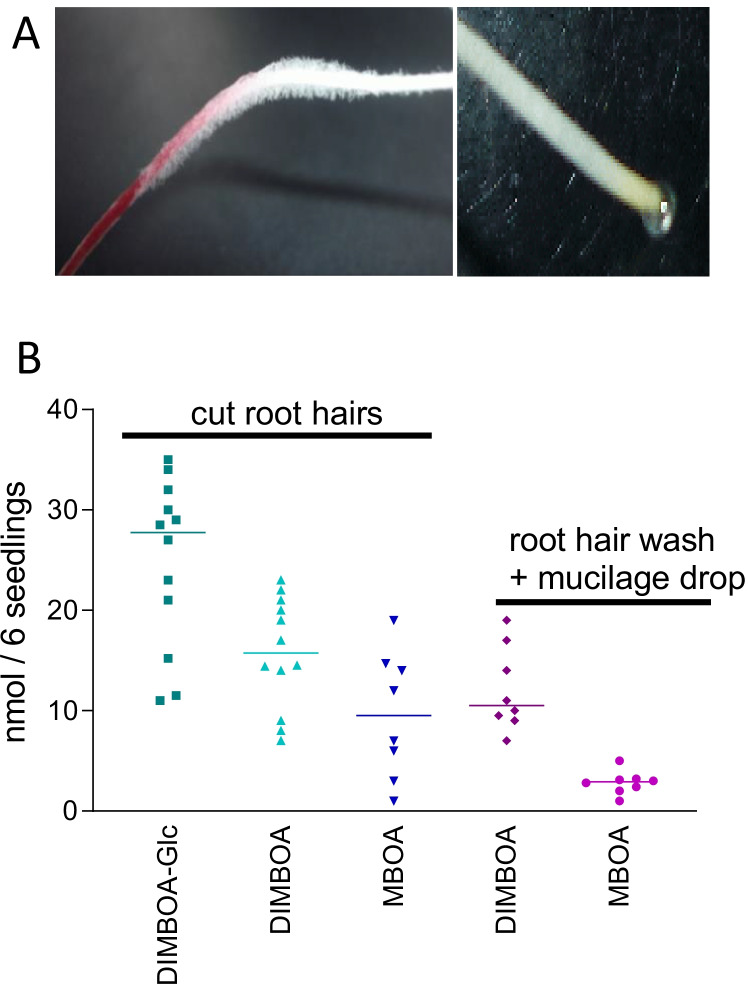
Maize root hairs (A) were cut and extracted. The extracts and washing solutions obtained from the root hair zone including the mucilage drop (B) were analyzed for benzoxazinoid contents. The extracts of cut root hair contain DIMBOA-glc, DIMBOA and MBOA (n = 12), the wash solution only DIMBOA and MBOA (n = 7) as major compounds

Emerging lateral roots destroy epidermal tissue, which leads only to a transient release of benzoxazinoids (Park et al. 2004). The major release of the compounds is therefore thought to take place by other mechanisms. Analyses of extracts from cut root hairs and of root hair zone wash solutions indicate that root hairs contain BXs and release the major portion of DIMBOA-glc/DIMBOA, either by active exudation or passively during root hair necrosis (Fig. 3). Indeed, a passive exudation of BXs during root hair necrosis is more likely, since specific inhibitors of membrane transporters did not suppress the release of BXs (Niculaes et al. 2018). Some detected MBOA indicated degradation. Amounts varied considerably (1 mg cut root hairs: 6.7–1.7 nmol DIMBOA-glc, 3.3 – 1.0 nmol DIMBOA and 3.0—0.2 nmol MBOA; root hair washing solution including mucilage drops/seedling: 1.1–1.2 nmol DIMBOA-glc, 1.8–4.2 nmol DIMBOA, 0.2–0.35 nmol MBOA). Neal et al. (2012) determined DIMBOA contents in exudates of 1 g fresh roots up to 31 µg after a 7 h collection. Although the data cannot be directly compared because of the different experimental design, the amounts released may be in a similar range. The identity of the compounds was ascertained by LC–MS/MS (DIMBOA-Glc: 372 (M-H)^−^, 396 (M + Na)^+^ 396 (6), 379 (4), 364 (3), 350 (4), 332 (3), 278 (4), 231 (48), 203 (25), 185 (100), 137 (8), 110 (33); DIMBOA: 212 (M + H)^+^, 234 (M + Na)^+^, 250 (M + K)^+^ 212 (5), 194 (8), 180 (9), 177 (34), 162 (32), 151 (36), 139 (18), 125 (8), 111 (100); MBOA: 164 (M-H)^−^, 166 (M + H)^+^, 188 (M + Na)^+^ 166 (100), 151 (45), 138 (8), 123 (10), 122 (25), 110 (63), 107 (17), 95 (5). BOA-6-OH or its glucoside was not detected in any of the samples. With this background, the chosen BOA-OH concentration of 500 µM for the subsequent experiments was thought to be high enough to elicit stress reactions in the root tip.

### Response of Maize Root Zones Exposed to BOA-OHs

**Fig. 4:**
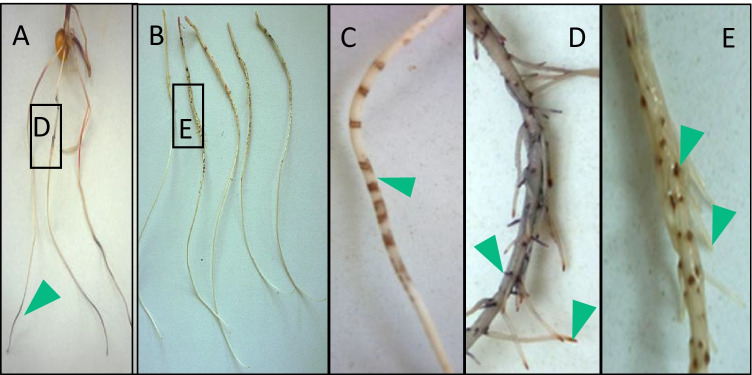
A, D: Maize root surface peroxidase assay (ABTS) exposes youngest root zones, the tips covered by the root cap and the bases of emerging roots as sites of peroxidase activity. B: Left to right: control, and after 24 h incubation with BOA-4-OH, BOA-5-OH, BOA-6-OH, BOA-7-OH. Dark polymers derived from BOA-OHs precipitate at lateral root emerging sites but not at the surface of root hairs or the tip, C: After BOA-OH incubations, ring-like structures developed preferentially at root zones prior to lateral root emergency. D: Root tips and root caps are dark after assaying peroxidase with ABTS. E: Dark spots at the emerging sites of lateral roots after BOA-OH incubations

**Fig. 5:**
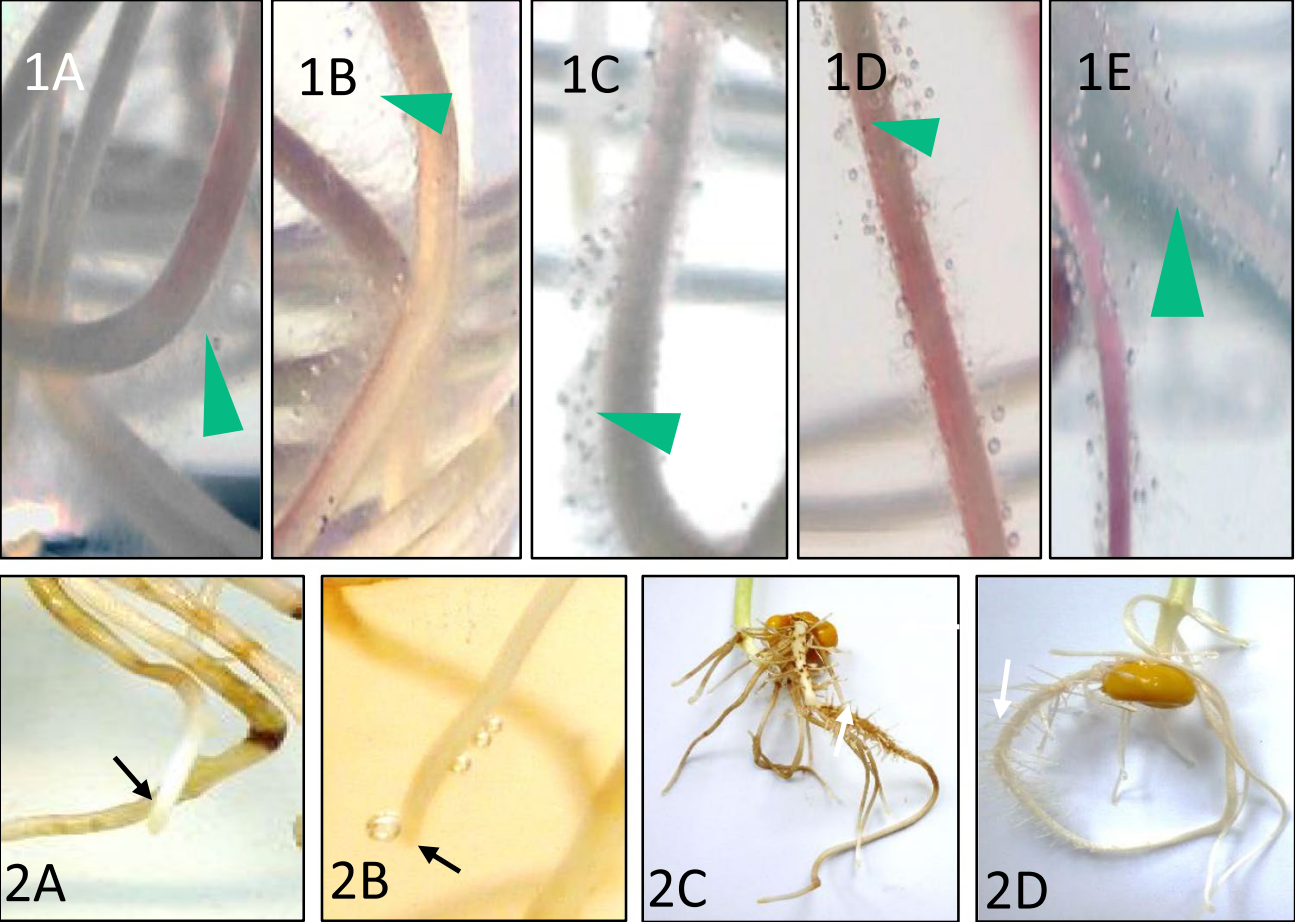
Reactions of maize roots. All BOA-OH isomers elicit weak to strong (shown here) bubble formation in the root hair zones with 0.5 mM BOA-OHs. 1A: Control, tap water + 500 µl MeOH; 1B: BOA-4-OH; 1C: BOA-5-OH; 1D: BOA-6-OH; 1E: BOA-7-OH. Arrow heads point to root hairs with bubbles at their tips and to root hairs of the control seedlings (1A). 2A: 500 µM of BOA-6-OH elicited no catalase activity developed during the first hours, arrow points to a white root tip (see gene expression study). 2B: Low catalase activity after 6 h; 2C: Whole plant to A2; 2D: Control plant

During incubation, the older parts of the maize roots developed increasingly dark brown spots and rings at areas where lateral roots emerged, whereas the tips stayed white. Browning started to get visible already during the first hr of incubation. The dark rings and spots indicate polymerization of the hydroxylated BOA-OHs most likely by peroxidases, as concluded from root surface peroxidase assays with ABTS (Fig. 4), but also polyphenol oxidase (PPO) and laccase activities cannot be excluded. Lateral root emergence is known to be accompanied by high peroxidase III abundance and H_2_O_2_/ROS production (Orman-Ligeza et al. 2016). Consequently, polymer deposition is especially high at the sites of lateral root emergence. The root hair zone and the root tip (root cap, meristem, and the beginning of the elongation zone) showed a different reaction compared to the older root parts with emerging and developed lateral roots. The root hair zone and the tip did not develop brownish spots or rings during the incubation with the BOA-OH isomers. The root hairs reacted with bubble formation shortly after the start of the incubation, which is indicative of H_2_O_2_ generation and its destruction by catalase, releasing O_2_. Destruction of H_2_O_2_ may prevent peroxidase-dependent polymer formation (Schellhorn and Stones 1992), (Fig. 5). Bubble formation at root hairs subsided during the course of incubation. Only a few bubbles developed at the root tips after 6 h of incubation.

The surfaces of maize root tips and the tips of lateral roots showed low peroxidase activity with the ABTS assay at root tips of control plants and those after BOA-OH exposure (Fig. 5). Correspondingly, BOA-OH incubated root tips and root hairs did not develop polymer coats at any time during the incubation. Thus, the root tips developed less visible reactions upon BOA-OH exposure than the root hairs (bubbles) or older root zones (polymers). Since root tips are known to be most sensitive to allelochemicals, membrane damage was assumed to occur. Therefore, we investigated effects on membrane lipids and fatty acids in maize root tips in comparison to *A. theophrasti* root tips after BOA-6-OH incubations, which was the major aim of this study.

### Effects of BOA-6-OH on Lipids

#### Total Unsaturated (UFA) and Saturated Fatty Acids

In higher plants, the most abundant fatty acids are oleic acid (18:1), linoleic acid (18:2), and α-linolenic acid (18:3). Under stress, the accumulation of free fatty acids in plant membranes is increased. Since unsaturated fatty acids (UFAs) are particularly susceptible to oxidation, we started with the determination of total UFAs in comparison to saturated fatty acid contents in the youngest (RTa) and older root tip parts (RTb).

**Fig. 6:**
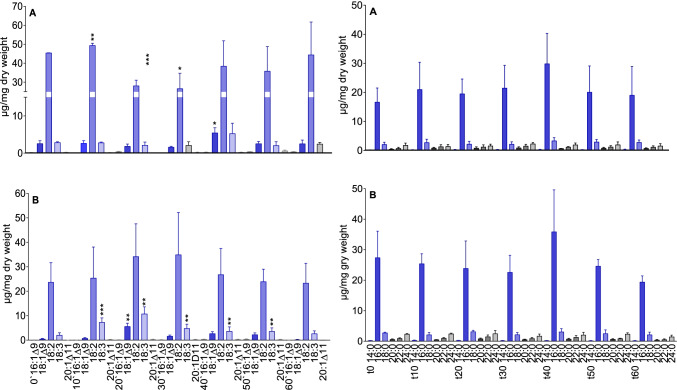
Total fatty acids in maize roots. Left side: unsaturated fatty acids; Right side saturated fatty acids. A: RTa (young root tissue), B: RTb (older root tissue). RTa und RTb samples were taken without (t0) and after exposure to 0.5 mM BOA-6-OH for 10, 20, 30-, 40-, 50- and 60-min. The figure shows mean values and standard deviations (n = 3). Significance with reference to t0 (t-test): *p < 0.05; **p < 0.005; ***p < 0.0005)

In the maize RTa and RTb samples, linoleic acid (18:2) is the main UFA, followed by α-linolenic acid (18:3), which is similar in amount as found for oleic acid. The analyses of the ZmRTa samples disclosed a significantly decreased free linoleic acid content 20 and 30 min after BOA-6-OH exposure. Regeneration of the t0 level was already accomplished after 40 min and at 60 min, no further decrease was observed. Except for a significant increase of oleic acid after 40 min, there was no other effect on α-linolenic acid and oleic acid contents during the entire course of incubation. In the ZmRTb samples, linoleic acid increased in tendency during BOA-6-OH exposure, but the effect was not significant. In contrast, α-linolenic acid increased significantly after 10 min and stayed constantly higher during the next 40 min, then dropped to the t0 value. During this period, also the amounts of oleic acid was higher. In both series, significant changes in the contents of saturated fatty acids were not detected (Fig. 6).

**Fig. 7:**
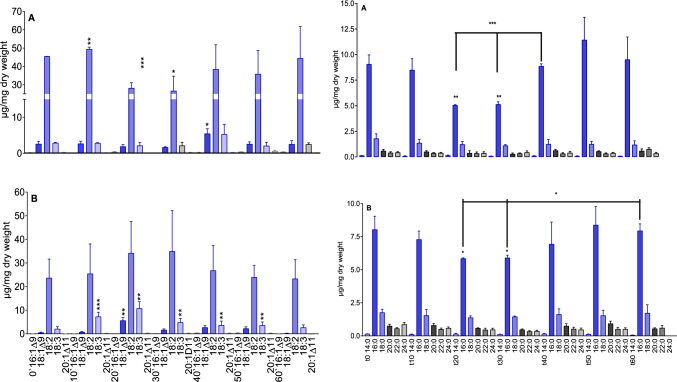
Total fatty acids in *A. theophrasti* root tips. Left side: unsaturated fatty acids; Right side: saturated fatty acids. A: RTa (young root tissue), B: RTb (older root tissue). RTa und RTb samples were taken without (t0) and after exposure to 0.5 mM BOA-6-OH for 10, 20, 30-, 40-, 50- and 60 min. The figure shows mean values and standard deviations (n = 3). Significance with reference to t0 (t-test): *p < 0.05; **p < 0.005; ***p < 0.0005)

In *A. theophrasti* RTa/RTb samples, linoleic acid and α-linolenic acid are the major total UFAs. With AbRTa, a significant decrease (approx. 50%) of both UFAs after 20 and 30 min was found. Their regeneration to t0 levels was reached after 40 min. A significant decrease of UFAs was observed in the AbRTb samples after 20 and 30 min. The subsequent regeneration was as found for AbRTa (Fig. 7). Major saturated fatty acids in *A. theophrasti* are palmitic acid (16:0) and stearic acid (18:0). Whereas stearic acid contents were not significantly affected, the ones of palmitic acid were reduced after 20 and 30 min of incubation with a regeneration to the t0 level after 40 min. The reduction was found in both sample series (AbRTa and AbRTb), with a stronger one in the youngest tissue, where a loss of about 50% of palmitic acid was measured. Thus, root tips of *A. theophrasti* are more affected than those of maize. Nevertheless, both species exhibit a remarkable fast regeneration of the fatty acid contents found at t0. The decrease of UFAs, and in case of *A. theophrasti* also the decrease of saturated fatty acids, is balanced already after 40 min.

**Fig. 8:**
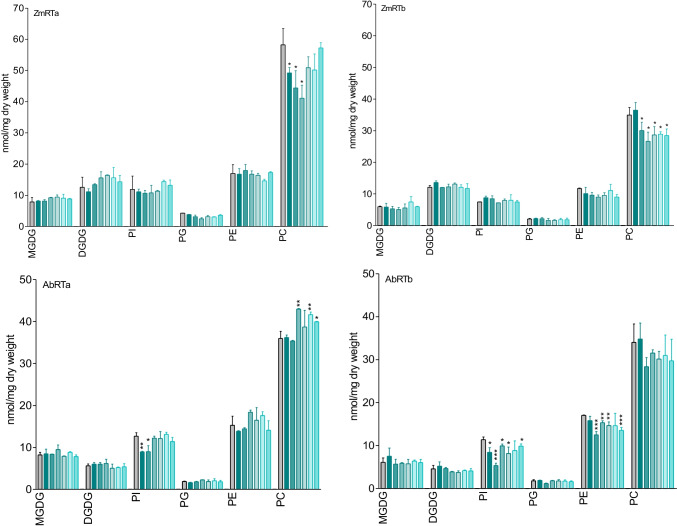
Maize (a:ZmRTa/b:ZmRTb) and *A. theophrasti* (a: AbRTa/b:AbRTb). Major structural phospholipids: PC phosphatidylcholine, PE phosphatidylethanolamine, PG phosphatidylglycerol, PI phosphatidylinositol. Glycolipids: MGDG, DGDG. Grouped columns present control and incubation times (left to right: 0 = control, 10, 20, 30, 40, 50, 60 min) of the different glyco/phospholipids. Molecular species are given in Fig. S1. Significance with reference to t0 (t-test): *p < 0.05; **p < 0.005; ***p < 0.0005)

For clues of structural phospholipid and glycolipid damage, their contents in the RTa/RTb series of both plants were determined and compared with the levels found in untreated roots set as t0 control (Fig. 8). In extra-chloroplast membranes, phosphatidylcholine (PC) and phosphatidyl-ethanolamine (PE) are the dominant constituents.

In the maize ZmRTa samples, only one of the major phospholipids, PC with the molecular species 34:2 and 34:4 (molecular species are shown in Fig. S1), was reduced during the first 30 min of BOA-6-OH incubation, then regenerated finally reaching t0 levels. PI and PG fluctuations followed the same trend, whereas DGDG, different from MGDG, had the trend to increase. However, these alterations were not significant. In the ZmRTb samples, only PC with the same molecular species as mentioned before, was affected, but in contrast to ZmRTa, the t0 level was not yet reconstituted after 1 h.

**Fig. 9:**
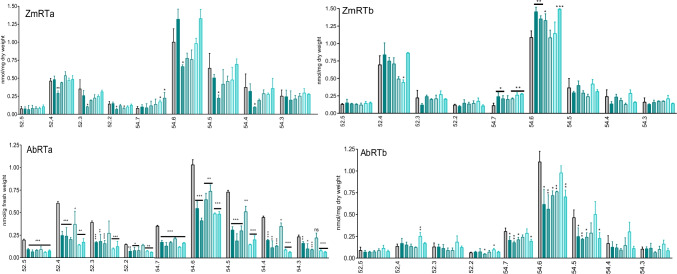
Maize TAGs: Series ZmRTa: A decrease in many molecular species is found after 20 min, while other species are not affected (52.5; 54.3) or increase (54.7). In ZmRTb, the major tendency was an increase of many species, although contents varied strongly. *A. theophrasti* TAGs: Series AbRTa: A decrease of most of the molecular species is found after 10 min. In AbRTb, the 52. 5–2 molecular species are less affected than the 54.7–3 ones. TAG 54.6 and 54.5 decreased during the first 20 min. Grouped columns present control and incubation times (left to right: 0 = control, 10, 20, 30, 40, 50, 60 min). Significance with reference to t0 (t-test): *p < 0.05; **p < 0.005; ***p < 0.0005)

In the *A. theophrasti* samples, galactoglycerolipid levels were not changed (Fig. 8). The most pronounced difference to maize was the significant decrease of phosphatidylinositol (PI) with the molecular species 34:3 and 34:2 in both sample series (AbRTa/b) during the first 20 min, followed by fast regeneration. In AbRTa, PC levels increased significantly after 30 min. Total PE content had a trend to increase after 20 and 30 min, but defined molecular species reacted differently (Fig. S1). For instance, PE 34:2 decreased during the first 20 min after the start of the incubation, while in ABRTb samples PE 34:2 levels were almost not affected, although total PE decreased (Fig. 8; S1). Total PC levels fluctuated without significance. However, regarding the molecular species, we found significant fluctuations with PC 34:3, 34:2, and 36:5. Thus, the responsiveness of phospholipids to BOA-6-OH was selective, depended on molecular species, and varied between the two plant species, with PC 34:2/34:4 as the significantly affected ones in maize and PI (34:3/34:2) in both *A. theophrasti* series. Finally, triacylglycerols (TAGs) and diacylglycerols (DAGs) contents were determined (Fig. 9). In the maize RTa samples, TAGs did not increase significantly above the t0 levels during BOA-6-OH exposure. Instead, a significant decrease, averaged to 30–40%, was found at t20 min with almost all molecular species (52.4; 52.3; 52.2; 54.6, 54.5, and 54.4), except for TAGs 52.5 and 54.7 which exhibited increasing levels. With all others, a subsequent regeneration to the t0 levels occurred. TAGs in maize RTb samples still fluctuated but in a different manner. The main molecular species 52.4 and 54.6, for instance, had a tendency to increase during the first 30 min, while decreasing later. To the end of the incubation after 60 min, both molecular species reached at least the level of t0 samples, in case of 54.6 higher ones.

In *A. theophrasti* RTa samples, a strong fluctuation of TAG molecular species was recorded with the 52.4, 52.3, 54.7, in particular with the 54.6, 54.5, 54.4, and 54.3 molecular species. The losses were highest in the 54.5 and 54.4 species (up to 50% and more). In the AbRTb samples, similar fluctuation patterns were found, with increases after 50 min in AbRTb samples, compared to shorter incubation times. TAG 54.6 and 54.5 decreased during the first 20 min. For most of the molecular species, the t0 levels were not reached again after 1 h, in contrast to maize RTa samples. TAG levels in most of the t0 samples of the RTa series were often higher than the ones found during the incubation, which may indicate that TAG levels decreased already very early during the incubation. The up and downs in the TAG profiles may be related to different and cascaded organized regeneration sites.

**Fig. 10:**
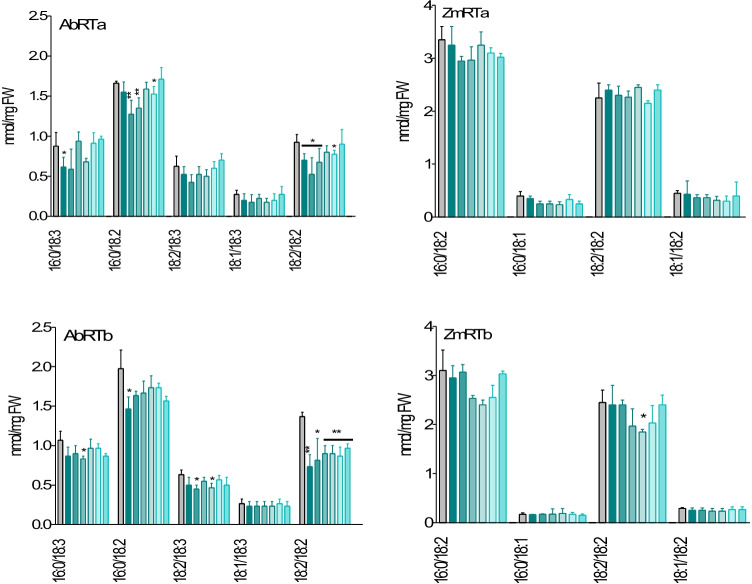
Defined DAGs are significantly affected in *A. theophrasti* (Ab) but not in maize (Zm) sample series RTa and RTb. Grouped columns present control and incubation times (left to right: 0 = control, 10, 20, 30, 40, 50, 60 min). Molecular species with low abundance (for instance 18:1/18:3 in Abutilon and 16:0/18:1 and 18:1/18:2 in maize are not affected. Significance with reference to t0 (t-test): *p < 0.05; **p < 0.005; ***p < 0.0005)

In maize RTa sample series, no alterations in the amounts of diacylglycerol (DAG) were found. In the ZmRTb sample series, DAGs, except for molecular species 18:2/18:2 at 40 min, were not significantly altered, although there was a tendency for reduction between 30–50 min. In the *A. theophrasti* RTa samples, the main DAGs showed slightly reduced levels during the early course of the incubation, but recovered later. In the older root part, an increase of the levels between 10 to 50 min was found for the DAGs 16:0/18:3 and 16:0/18:2. In most of the *A. theophrasti* samples, DAG t0 levels were higher, however predominantly without significance. For both species, DAGs were less affected than TAGs. Since DAGs of *A. theophrasti* roots exhibited some responsiveness, PA values were here determined. PA amounts were only enhanced after 20 min in series a, and after 10 min in series b, otherwise not significantly affected (Fig. 10; Fig. S2).

The lipid analyses disclosed 1) an early decrease of defined membrane phospholipids, from which PI in *A. theophrasti* and PC in maize are most affected with defined molecular species. 2) The highest response was ascertained for total fatty acids, particularly the decrease of UFA 18:2 in the youngest tissue in both species. UFA 18:3, not affected in ZmRTa samples, increased in ZmRTb samples, but considerably decreased in both *A. theophrasti* series. Palmitic acid was only reduced in *A. theophrasti*. 3) Regarding the neutral lipids, TAGs were affected in both species, but DAGs were almost not changed in maize, and *A. theophrasti* to a much lower degree than TAGs. 4) Disturbances of membrane PL contents were rapidly balanced in young root tissue of both species. The data indicate a) different PLs with defined molecular species as major targets for BOA-6-OH damage and b) differences in membrane repair of maize and *A. theophrasti*.

### Gene Expression in Maize Root Tips

**Fig. 11:**
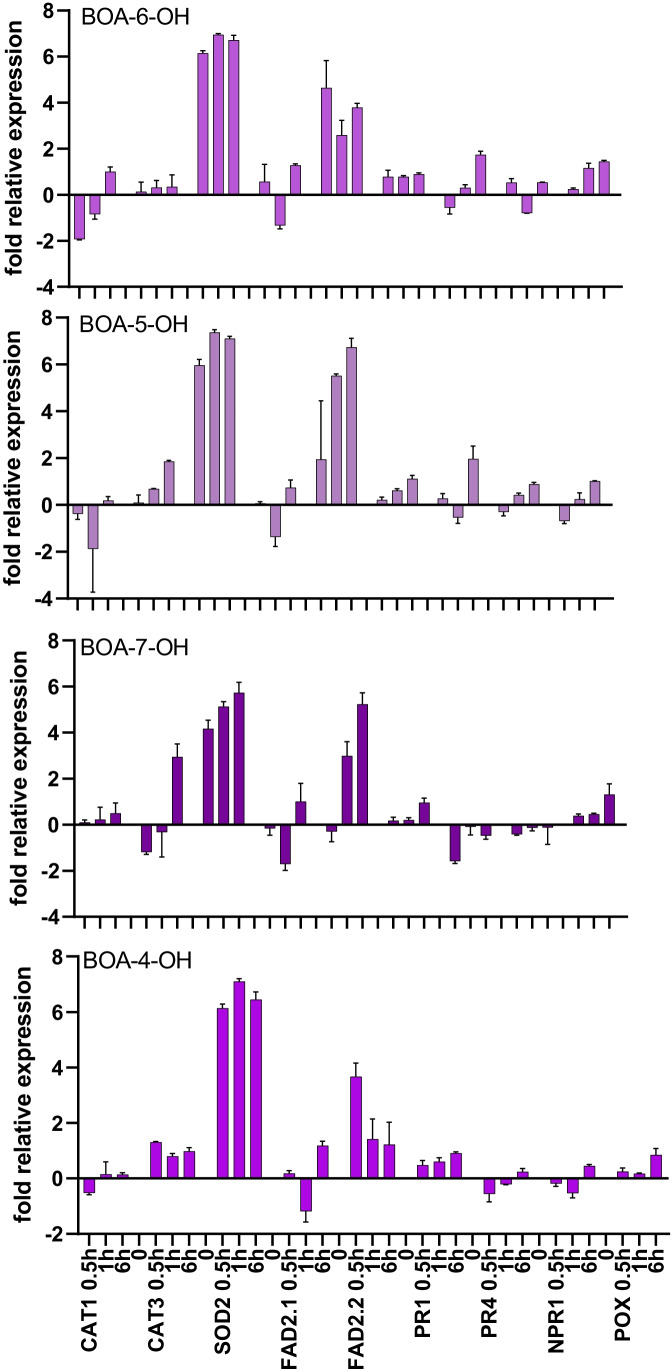
Maize genes affected by BOA-OH isomers. Relative transcript abundance of genes encoding enzymes involved ROS detoxification (SOD2; CAT1, CAT3), fatty acid desaturation (FAD2.1; FAD2.2) and genes related to pathogenesis and resistance (PR1, PR2, NPR1, POX12), mean values ± STD, n = 3, shown as fold-changes (log_2_^− ΔΔCt^). Responses to BOA-OH isomer exposure are shown for 30 min, 1 h and 6 h incubation times

Gene expression studies were performed by qRT-PCR to unravel whether ROS caused oxidative stress and the changes in lipid composition were based on transcriptional regulation. The expression of *SOD2* was upregulated in the root tip when the seedlings were exposed to the BOA-OHs, shown in Fig. 11 for the 30 min to 6 h incubation period. Except for BOA-7-OH, all isomers elicited a 6-sevenfold higher *SOD2* expression after 30 min of incubation. The high levels were maintained over a period of 6 h, then dropped to 4–4.5fold after 24 h. With BOA-7-OH, a retarded increase to a similar extent was found (6.5fold after 24 h).

The *FAD2-2* gene expression during BOA-6-OH exposure was increased after 30 min up to more than fourfold and maintained at higher levels for at least 6 h. The up regulation of *FAD2-2* expression was higher with BOA-5-OH isomers and reached a sevenfold expression after 6 h (BOA-5-OH, thus this isomer may cause more severe lipid damage. In contrast, *FAD2-1* had low responsiveness, similar to *CAT1*. *CAT3* expression was slightly enhanced with BOA-4-OH and BOA-6-OH, but it was increased with BOA-5-OH by twofold after 6 h. An almost threefold increase was found after 6 h with BOA-7-OH.

*NPR1* and *POX12,* two genes involved in regulation of pathogenesis and resistance, were not responsive. *PR4* was about twofold up regulated after a 6 h exposure to BOA-6-OH and BOA-5-OH. *PR1* was up regulated after 24 h in presence of BOA-4-OH (1.8fold) and of BOA-7-OH (3.2fold). Thus, the expression profiles of *CAT3, PR4,* and *PR1* seem to be specific for the isomers, while the abundant naturally occurring BOA-6- and BOA-5-OH had similarities in their effects. The response may be specific for the concentration and presumably dependent on the duration of exposure.

### Accumulation of BOA-OH Detoxification Products

Maize seedlings started to glucosylate BOA-6-OH almost immediately when exposed to the compound. Within 10 min, about 100 nmol/g FW of the glucosides were produced in the root tips. The amounts of the detoxification product increased constantly further on, reaching about 500 nmol/g FW within 1 h.

**Fig. 12:**
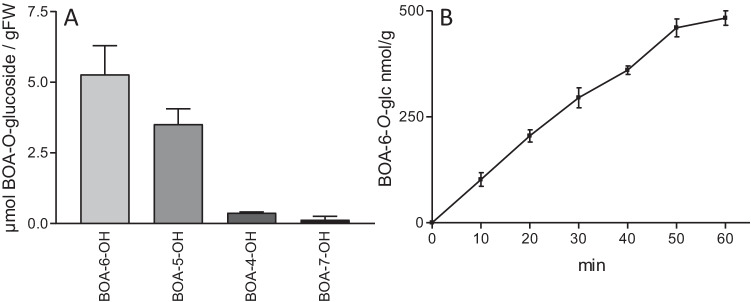
A: Contents of detoxification products in maize roots exposed to BOA-OHs for 24 h. HPLC analysis of methanolic extracts from maize roots reveals the accumulation of BOA-6-O-glucoside and BOA-5-O-glucoside, but almost no products of BOA-4-OH and BOA-7-OH. B: BOA-6-O-glc accumulation in root tips during the first h of incubation

For the long-term accumulation of detoxification products, also the other isomers were tested. Within 24 h, ~ 5 µmol/g FW BOA-6-*O*-glucoside and ~ 3 µmol BOA-5-*O*-glucoside accumulated in the roots, but only traces of not further analyzed BOA-4-OH and BOA-7-OH detoxification products (Fig. 12). The fast glucosylation of BOA-6-OH, concomitant with ROS elimination, is supposed to be essential for blocking continued membrane damage, thereby explaining the lower up-regulation of *FAD2-2*. Analyses of the incubation media after 24 h revealed a strong decrease of BOA-6-OH and BOA-5-OH to about 5% and 9% of the original amounts, and also of BOA-4-OH (3%) and BOA-7-OH (6%), indicating that additional mechanisms contribute to the compounds´ detoxification or elimination by polymerization. The position of the OH group strongly influenced the detoxification via glucosylation.

## Discussion

### Maize Root Zone Dependent Reactions

The early defense strategy against allelopathic BOA-OHs encompasses contemporaneous ROS and BOA-OH detoxification and lipid regeneration in the youngest root zones of maize seedlings whereas older root zones with emerged lateral roots predominantly performed compound polymerizations at the surface. We strongly assume that root surface colonizing microorganisms contribute to ROS detoxification, i.a. by catalase activity. When freshly collected microorganisms from the maize root were exposed to BOA-OH isomers, they reacted with bubble formation. However, the microorganisms are not yet identified and their contributions need further investigation. In a former study was shown, that *A. theophrasti* developed bubble formation as a response to BOA-OH treatment which was, however, not restricted to root hairs, but covered the entire root during the early phase of incubation (Schulz et al. 2018). From these experiments, we conclude that not only the plants but also the microorganisms suffer from the allelochemicals and start defense and detoxification reactions. The root zone-dependent reaction of maize could be severely influenced by specific microbial assemblies, characteristic of the defined root zone (Bonkowski et al. 2021). The density of microbial colonization and the species diversity within established microbiomes are described to be root zone dependent (Massalha et al. 2017; Schmidt et al. 2018; Rüger et al. 2021). Variations in the species composition on root surfaces are also heavily influenced by the soil. For instance, colonization of *A. theophrasti* seeds with a natural *Actinomucor elegans* consortium depends on the soil microbiome of the cultivation site. Seedlings grown from these seeds developed another detoxification behavior for BOA-OHs (Schulz et al. 2018). Root hair colonizing microorganisms, such as beneficial *Pseudomonas* species, are known from *Arabidopsis*, rice, tomato, and maize (Prieto et al. 2011), and many of these microorganisms possess catalases (Kim and Park 2014). The different reactions towards BOA-OH isomers depend therefore on root zone characteristics in combination with responses of defined microorganisms that may shield the root from fatal allelochemical injuries. Until these mechanisms come into effect, minimization of cellular damage requires further, plant-specific defense reactions, in particular in the vulnerable root tips which have not yet mature microbial assemblies, well-coordinated in their functions.

Aside from polymerizations or glucosylations, elimination of the BOA isomers may probably occur by additional mechanisms. Although radical scavenger functions of many phenolic compounds are a common assumption, mixtures of exogenous phenolics have been found to elicit an oxidative burst-like reaction (Baker et al. 2005; O'Brien et al. 2012).

Benzoxazinoids released from root hairs and applied BOA-OHs may undergo co-oxidation, which can result in compound destruction. This mechanism could contribute to BOA-OH elimination and may explain the strong reduction of BOA-OH contents from the mediums after 24 h incubations. Perhaps, root hairs and maize root tips are therefore not covered with polymer coats.

### Maize Gene Inductions, BOA-OH Glucosylation, Lipid Damage and Repair

BOA-OH isomers elicited a fast relative increase of *SOD2* transcripts in maize root tips for superoxide anion radical detoxification. *SOD2* expression was upregulated within 30 min by all BOA-OH isomers, indicative of a fast elevation of the ROS levels in the young root cells. The alteration of relative *SOD2* transcript abundance is much faster than found in maize cultivars infested with aphids, which led to a transcript elevation from 1.3 to 5.4fold after 4 to 8 h (Sytykiewicz 2014). *SOD2* gene induction is also responsive to paraquat and the allelochemical juglone (Gail et al. 1986).

Whereas *CAT1* transcript abundance was not changed, *CAT3* gene expression was weakly enhanced after 6 h incubation with the BOA-5/7-OH isomers. The induction of *CAT3* gene expression was congruent with a weak bubble development at the root tip surface after 6 h of incubation with BOA-6-OH. In juglone treated germinating maize kernels, none of the catalase genes was induced after 24 h, even when 1 mM juglone was applied. An increase in catalase activity after ferulic acid application was observed in maize roots but only with concentrations higher than 1 mM (Rama Devi and Prasad 1996). Cruz-Ortega et al. (2002) found a more than 50% decreased catalase activity in maize roots after exposure to an aqueous leachate of allelopathic *Callicarpa acuminata.* We waived measurements of catalase activity, because it is not possible to distinguish between plant and microbe derived activities in crude protein extracts. From our results, we conclude that CAT1 is not, and CAT3 arguably integrated at a later stage of the detoxification process, but both enzymes do not seem to be responsible for the major H_2_O_2_ elimination during the first hours of BOA-OH exposures. We hypothesize that the major part of catalase activity is attributed to root colonizing microorganisms. Indeed, we can presently not exclude BOA-OH stressed microorganisms as the earliest and main producers of ROS, that enter root cells prior to catalase induction and which are mainly responsible for the almost immediate damage of membrane lipids in the youngest root tissue.

*SOD2* gene expression is concomitant with elevated expression of desaturase *FAD2-2*. FAD2-2 is the major oleoyl desaturase responsible for the synthesis of linoleic acid, a constituent of plant membranes (Hernández et al. 2009), thus the ER membrane-bound FAD2-2 enzyme is essentially important for the regeneration of the linoleic acid (18:2) pools in maize root tips after BOA-OH exposure. Linoleic acid is the most affected UFA during BOA-6-OH incubations. FAD2 and FAD3 (not measured) are primarily responsible for the desaturation of extra-chloroplastic phospholipids. They may particularly contribute to fast membrane repair. FAD2 converts oleate bond to phosphatidylcholine into linoleate by introducing a sec double bond at the C12 position. Whereas it is known that cold stress induces enhancement of the *FAD2-2* gene expression for membrane adaptation (Dar et al. 2017), we could not find reports on *FAD2-2* transcript enhancement caused by chemical stress. *FAD2-1* and *FAD2-2* are isogenes and their expression is differently regulated, as concluded from their co-expression networks with different transcription factors in maize (Zhao et al. 2019), which may orchestrate their responsiveness to (allelo)chemical stress.

Clearly, BOA-OH isomers elicit ROS and damage to membrane lipids. Ingólfsson et al. (2014) emphasized cell membrane perturbations by altered bilayer properties due to phenolic phytochemicals getting attached to the bilayer/solution interface. The compounds merge to the membrane by hydrophobic interaction, resulting in softened, partitioned phospholipid bilayers that influence membrane proteins functions and activities. Bilayer destabilization and alteration of membrane fluidity, also found for the allelochemical sorgoleone (Lebecque et al. 2019), are the consequences, not only in plants but probably also in microbial membranes. We hypothesize that these perturbations activate lipases and perhaps lipoxygenases to remove affected fatty acids from membrane phospholipids for fatty acid exchange and reestablishing correct membrane functioning. Presumably, the removal of fatty acids that are in contact with BOA-6/5-OH is closely linked to the glucosylation step for detoxification which may present a prerequisite for membrane repair. The presence of UGT for glucosylation already in the cell wall, as was found for maize UGT BX9, is reasonable for protecting the plasma membrane first, having higher contents of unsaturated phospholipids in the cytosolic leaflet, and later other membranes in the cell. Since BOA-4-OH and BOA-7-OH do not accumulate as glucosides, and BOA-5-OH to a lower degree, oxidative stress, and membrane damage may be higher with these isomers, while the effects of BOA-7-OH are retarded due to the low solubility. In addition, maize may be adapted to the occurrence of BOA-6-OH in the cell wall.

TAGs seem to be affected mainly in the youngest root part in both plant species, but due to the strong variations during the course of incubation, it is presently difficult to interpret the role of BOA-6-OH on TAGs. In maize, only the contents of a few molecular species were changed, in contrast to *A. theophrasti*. The most abundant molecular species 56:4 is affected in both species, while others show a weaker response.

As mentioned above, TAGs, which are compartmented in lipid droplets, serve mainly as storage compounds. In vegetative tissues and under normal growth conditions, only a few lipid droplets are found, while during stress conditions, such as drought, the amounts of TAGs are increased. In plants and animals, the increase in TAG production is generally considered as a detoxification reaction to lower the concentrations of free fatty acids to avoid lipotoxicity (Piccolis et al. 2019). An increase of lipid droplets and TAGs is described to occur during long term adverse conditions elicited by nutrient, light, heat, cold, and salt stresses. Here, it can be hypothesized that fatty acids can be released from TAGs for membrane lipid repair, and TAGs may have a role in maintaining fatty acid homeostasis. TAGs from lipid droplets are thought to function as precursors for membrane re-synthesis (Lee et al. 2020). In yeast, storage lipid synthesis and membrane biogenesis have been described to be coordinated (Gaspar et al. 2011). TAGs may be released also for energy homeostasis during metabolic conditions demanding high energy inputs, such as stress conditions. A decrease in TAGs to raise the energy costs necessary for counteracting membrane and other damages, elicited by allelochemicals of the dinoflagellate *Alexandrium minutum,* was also reported for the diatom *Chaetoceros muelleri* (Long et al. 2018). According to our results, TAGs may function in this way in short term BOA-OH stressed maize and *A. theophrasti* root tips, allowing a fast repair within 20 to 40 min of damaged structural phospholipids. DAGs, released from TAGs, could serve as building blocks for PE and PC synthesis (Henry et al. 2012). A dissection of the complex interactions and BOA-OH impacts on TAG metabolism clearly requires further investigations.

Regarding phospholipid alterations, we found differences between maize and *A. theophrasti*. Whereas in maize PC was the only phospholipid that was significantly reduced during the first 30 min in the youngest part of the root tip, PI was the most affected phospholipid in both *A. theophrasti* RTa and RTb samples. The decrease of PI could indicate a short-term activation of the phosphoinositide-specific phospholipase C signaling pathway, important for plant immunity (Vossen et al. 2010), which does not occur in maize. For verification, further studies are required. Also, other phospholipases are activated by H_2_O_2_, such as phospholipase D which releases phospholipid head groups (Yamaguchi et al. 2004). Since the membrane repair mechanisms in maize and *A. theophrasti* are highly efficient, further downstream events, for instance continuing release of signaling molecules, do not occur in critical amounts.

### Connection to Immune Response

The efficient elimination of successive H_2_O_2_ by SOD2 and probably microbial catalases together with the fast repair of membrane lipids should diminish further oxidation of C18 UFAs. Thus, an increase of lipoxidation end-products from UFA 18:3, leading to the jasmonate precursor 12-oxophytodienoic acid (OPDA), might be suppressed in maize but perhaps not in *A. theophrasti* to a similar degree. According to Liu et al. (2016), genes related to ROS production and scavenging systems, for instance, catalase and superoxide dismutase, are not responsive to methyl jasmonate treatment.

The oxidized fatty acid derivatives *cis*-OPDA and jasmonate have, however, a pivotal role in plant defense (Schenk and Schikora 2015). NPR1 has an important function in systemic acquired resistance (SAR) and induced systemic resistance (ISR) by triggering cross-talk between the salicylic acid (SA) and JA (Backer et al. 2019). The transcription of *NPR1* is positively influenced by the SA pathway that stimulates transcription of *PR1*, *PR2* and, *PR5*, leading to systemic acquired resistance (SAR), (Ali et al. 2018). *POX 12*, belonging to the SA markers, is not induced. Expression of antifungal *PR1*, as one member of this PR group, and *NPR1* showed no strong response elicited by the BOA-OH isomers within 6 h, but BOA-4-OH and BOA-7-OH induced a moderate up regulation after 24 h. PR4, an antifungal chitinase II, enhances resistance against biotrophic and necrotrophic fungal phytopathogens. *PR4* transcription is up regulated by the JA defense signaling pathway which activates local acquired resistance (LAR). Only the most abundant naturally occurring BOA-6-OH and BOA-5-OH led to a low enhancement of *PR4* expression after 6 h, indicating that linolenic acid derivatives could be too low to stimulate the JA defense signaling pathway efficiently. We believe that JA precursors from nonenzymatic peroxidation of linolenic acid do not accumulate in sufficient amounts to trigger plant defense reactions considerably. On the other hand, cleavage of linoleic acid (18:2) or linolenic acid (18:3) from membrane lipids can submit to pathways resulting in so-called death acids (Christensen et al. 2015), which have also special roles in defense and stress responses. According to our results, membrane damage by the allelochemicals, resulting in oxidized fatty acids, may be eventually linked to key events important for plant immune responses after long term application, but presently, the molecular events explaining the linking are elusive.

## Supplementary Information

Below is the link to the electronic supplementary material.

Supplementary file1 (DOCX 257 KB)

## Data Availability

Data sets for archives were not generated. The data sets generated during and/or analysed during the current study are available from the corresponding author.
