# Supplementary material for: Survival of Plants During Short-Term BOA-OH Exposure: ROS Related Gene Expression and Detoxification Reactions Are Accompanied With Fast Membrane Lipid Repair in Root Tips
Source: J Chem Ecol. 2022 Jan 5;48(2):219–39. doi: 10.1007/s10886-021-01337-z (PMC8881443; doi:10.1007/s10886-021-01337-z)
Supplement: Supplementary file 1 — Supplementary file1 (DOCX 257 KB) [file 10886_2021_1337_MOESM1_ESM.docx]

**Supplementary Information (SI)**

**SURVIVAL OF PLANTS DURING SHORT-TERM BOA-OH EXPOSURE: ROS RELATED GENE EXPRESSION AND DETOXIFICATION REACTIONS ARE ACCOMPANIED WITH FAST MEMBRANE LIPID REPAIR IN ROOT TIPS**

LAURA LASCHKE^1,4^, VADIM SCHÜTZ^1,4^, OLIVER SCHACKOW^2^, DIETER SICKER^2^, LOTHAR HENNIG^2^, DIANA HOFMANN^3^, PETER DÖRMANN^1^and MARGOT SCHULZ^1^*

*^1^IMBIO Institute of Molecular Physiology and Biotechnology of Plants, University of Bonn, Karlrobert-Kreiten Str. 13, 53115 Bonn, Germany*

*^2^Institut für Organische Chemie, Universität Leipzig, Johannisallee 29, 04103 Leipzig, Germany*

*^3^IBG-3: Agrosphäre, Forschungszentrum Jülich GmbH, Jülich, Germany*

^4^Authors contributed in experimental work equally

Corresponding author: Margot Schulz, [ulp509@uni-bonn.de](mailto:ulp509@uni-bonn.de)

orcid.org/0000-0001-7373-306X

**
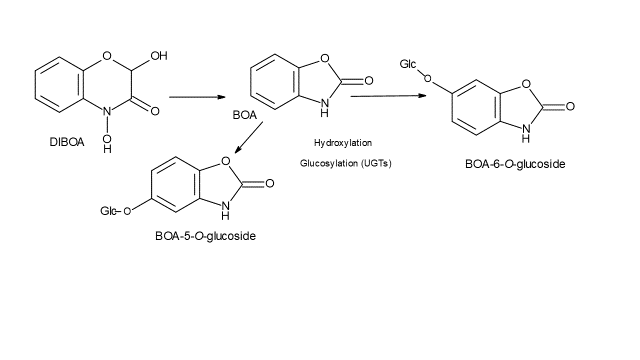
**

**Figure 1S:** Degradation of **2,4-Dihydroxy-2H-1,4-benzoxazin-3(4H)-one (DIBOA) to** 2-benzoxazolinone (BOA). Plants hydroxylate BOA either in position 5 or 6. The BOA-OHs are subsequently glucosylated. The glucosides present BOA detoxification products.


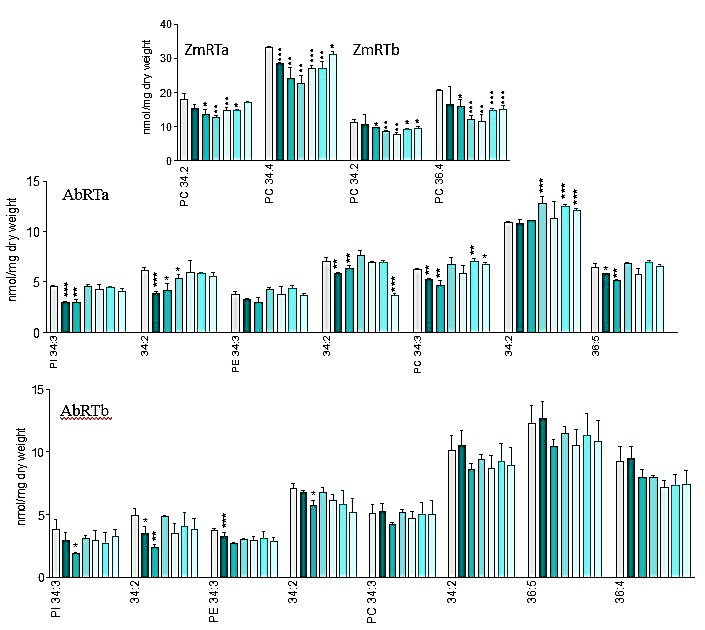


**Figure 2S:** Affected and not affected molecular species of major phospholipids in RTa (young root tip tissue) and RTb (older root tip tissue) samples of maize (Zm, above left RTa, right RTb) and *Abutilon theophrasti* (Ab, middle AbRTa, below AbRTb). Grouped columns present control and incubation times (left to right: 0 = control, 10,

20, 30, 40, 50, 60 min). PLs: phosphatidylcholine (PC), phosphatidylethanolamine (PE), phosphatidylinositol (PI).

**Figure 3S**: Phosphatidic acid generated with low significance only in *A. theophrasti* after 20 min (RTa PA1) and 30 min (RTb PA2) of incubation.

**SI Table S1**: Primers used in the study

| **Gene** | **Primer Sequence** |
| --- | --- |
| Cat1 forw. | ACCTGAAGCCGAGCATGTAAGGAT |
| Cat1 rev. | ATAATCGACCACCGACCATCAGCA |
| Cat3 forw. | CCGGCTCAACATGAAGGCAAACAT |
| Cat3 rev. | TTCTCTTGTTCCTGGCGACGACAT |
| FAD2.1 forw. | ACCACCTCTTCTCCACCATGCC |
| FAD2.1 rev. | CCTTGCGGTTGCGGTTCTCAG |
| FAD2.2 forw | TGCCGCTGCTGATCGTGAAC |
| FAD2.2 rev. | TGCGTGTCCGTGATGTTGTGG |
| NPR1 forw. | CTCCAGAGGGGCACAGCCGA |
| NPR1 rev. | CGAGAACACGCTGCCCTCCG |
| PR1 forw. | TCAGTCATGCCGTTCAGCTT |
| PR1 rev. | TTGTCCGCGTCCAGGAA |
| PR4 forw. | TGATGGATAGATGGCGATTGC |
| PR4 rev. | AGAATTGACACCGCCAAACC |
| Sod2 forw. | CACCAACGGCTGCATGTC |
| Sod2 rev. | ATGCTCCTTGCCAACAGGAT |
| Act1 forw. | GGGATTGCCGATCGTATGAG |
| Act1 rev. | GAGCCACCGATCCAGACACT |
